# NADPH oxidase and endoplasmic reticulum stress is associated with neuronal degeneration in orbitofrontal cortex of individuals with alcohol use disorder

**DOI:** 10.1111/adb.13262

**Published:** 2022-12-10

**Authors:** Liya Qin, Ryan P. Vetreno, Fulton T. Crews

**Affiliations:** ^1^ Bowles Center for Alcohol Studies, School of Medicine University of North Carolina at Chapel Hill Chapel Hill North Carolina USA; ^2^ Department of Psychiatry, School of Medicine University of North Carolina at Chapel Hill Chapel Hill North Carolina USA; ^3^ Department of Pharmacology, School of Medicine University of North Carolina at Chapel Hill Chapel Hill North Carolina USA

**Keywords:** cell death, cerebral cortex, endoplasmic reticulum stress, human, oxidative stress

## Abstract

Many disorders of the central nervous system (CNS), including alcohol use disorder (AUD), are associated with induction of proinflammatory neuroimmune signalling and neurodegeneration. In previous studies, we found increased expression of Toll‐like receptors (TLRs), activated NF‐κB p65 (RELA), and other proinflammatory signalling molecules. Proinflammatory NADPH oxidases generate reactive oxygen species, which are linked to neurodegeneration. We tested the hypothesis that AUD increased RELA activation increases NADPH oxidase‐oxidative stress and endoplasmic reticulum (ER) stress cell death cascades in association with neuronal cell death in the human orbitofrontal cortex (OFC). In the AUD OFC, we report mRNA induction of several NADPH oxidases, the dual oxidase *DUOX2*, and the oxidative stress lipid peroxidation marker 4‐HNE and the DNA oxidation marker 8‐OHdG that correlate with RELA, a marker of proinflammatory NF‐κB activation. This was accompanied by increased expression of the ER stress‐associated regulator protein glucose‐regulated protein 78 (GRP78), transmembrane sensors activating transcription factor 6 (ATF6), protein kinase RNA‐like endoplasmic reticulum kinase (PERK), and inositol‐requiring kinase/endonuclease 1 (pIRE1), and the pro‐apoptotic transcription factor C/EBP homologous protein (CHOP). Expression of NADPH oxidase‐oxidative stress markers correlate with ER stress‐associated molecules. Induction of oxidative stress and ER stress signalling pathways correlate with expression of cell death‐associated caspases and neuronal cell loss. These data support the hypothesis that proinflammatory RELA‐mediated induction of NADPH oxidase‐oxidative stress and ER stress‐associated signalling cascades is associated with neuronal cell death in the post‐mortem human OFC of individuals with AUD.

## Graphical abstract

Alcohol use disorder (AUD) induces proinflammatory oxidases in the human orbitofrontal cortex (OFC) in association with upregulation of proinflammatory RELA, consistent with NF‐κB neuroimmune signaling contributing to oxidative stress. Induction of oxidative stress is correlated with induction of endoplasmic reticulum (ER) stress‐associated signaling cascades and activation of cell death‐associated caspases and neuronal loss. These data implicate neuroimmune signaling and oxidative stress in induction of ER stress as contributing to neuronal death in the OFC of individuals with AUD.

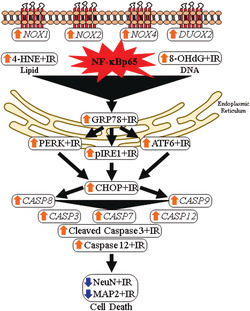

## 1 INTRODUCTION

Neuroimmune signalling and neuronal cell death are common features of alcohol use disorder (AUD).^1^, ^2^, ^3^ Neuroimmune signalling molecules are expressed in the human brain and thought to contribute to neuronal cell death,^3^, ^4^, ^5^, ^6^, ^7^ but the mechanism remains to be fully identified. The orbitofrontal cortex (OFC), which is critically involved in regulating executive function,^8^ is vulnerable to damage in human AUD. Significant neuronal loss, volumetric reductions, and decreased connectivity are reported in the OFC of individuals with AUD and are accompanied by deficits in executive functioning.^1^, ^9^, ^10^, ^11^ In a prior study, we reported a link between AUD‐associated upregulation of Toll‐like receptors (TLRs), the cytokine‐like endogenous TLR agonist high‐mobility group box 1 (HMGB1), and downstream activation of the proinflammatory nuclear transcription factor NF‐κB p65 (RELA) with neuronal cell death in the post‐mortem human OFC.^3^ In addition to transcription of proinflammatory cytokines and chemokines,^3^, ^12^, ^13^, ^14^ RELA nuclear transcription induces proinflammatory nicotinamide adenine dinucleotide phosphate (NADPH) oxidases and generation of reactive oxygen species (ROS).^1^, ^15^ Preclinical studies implicate NADPH oxidases and generation of ROS in ethanol‐induced neuronal cell death.^1^, ^16^ With the exception of NOX2 (i.e., gp91PHOX^1^), the role of other NADPH oxidases, dual oxidases (DUOX), and oxidative stress in AUD‐induced neuronal cell death in the human OFC has not been clearly defined, promoting assessment in the current study.

NADPH oxidase produces superoxide, a major source of cellular ROS that can cause endoplasmic reticulum (ER) stress.^17^ Oxidative stress‐induced disruption of the reduction–oxidation (REDOX) balance impairs protein folding and processing, leading to accumulation of unfolded and misfolded proteins in the ER triggering the unfolded protein response (UPR).^18^, ^19^, ^20^ The UPR involves dissociation of glucose‐regulated protein 78 (GRP78) from ER resident transmembrane sensor proteins, including inositol‐requiring kinase/endonuclease 1α (IRE1α), protein kinase RNA‐like endoplasmic reticulum kinase (PERK), and activating transcription factor 6 (ATF6). This leads to restoration of ER homeostasis^21^, ^22^ through degradation of ER‐bound mRNAs, inhibition of protein translation, and formation of protein folding enzymes.^23^, ^24^ While transient UPR can typically enhance the protein folding capacity of the ER to overcome ER stress,^25^ chronic long‐lasting ER stress that does not recover results in cellular dysfunction and cell death through activation of the pro‐apoptotic transcription factor C/EBP homologous protein (CHOP).^26^, ^27^ In turn, CHOP activation promotes transcription of pro‐apoptotic cell death genes, such as caspases.^28^ In neuronal cell culture, acetaldehyde, the main toxic metabolite of alcohol, dose‐dependently induces ROS generation, upregulates GRP78‐ATF6, pIRE1, and PERK‐CHOP signalling cascades, and induces caspase 3/9/12‐mediated cell death that is inhibited by treatment with the anti‐oxidant *N*‐acetyl‐l‐cysteine and the anti‐inflammatory HDAC inhibitor sodium phenylbutyrate.^29^ While ER stress has been implicated in human alcoholic liver disease (ALD) and investigated in preclinical models of ALD,^30^, ^31^ the association of AUD‐induced NADPH oxidase‐oxidative stress and ER stress with neuronal cell death in the human OFC is unknown. This led us to test the hypothesis that AUD‐associated neuroimmune signalling through RELA induces NADPH oxidase‐oxidative stress and ER stress‐signalling cascades in association with the observed neuronal cell death in the human OFC.

## 2 MATERIAL AND METHODS

### 2.1 Human tissue

Post‐mortem human male OFC paraffin‐embedded and frozen tissue samples from moderate drinking control (CON) and individuals with AUD (*n* = 10/group) were obtained from the New South Wales Brain Tissue Resource Centre (NSW BTRC [Ethnics Committee Approval Number: X11‐0107]) at the University of Sydney (supported by the National Health and Medical Research Council of Australia‐Schizophrenia Research Institute and the National Institute of Alcohol Abuse and Alcoholism [NIH (NIAAA) R24AA012725]). Individual information was collected through personal interviews, next‐of‐kin interviews, and medical records and is presented in Table 1. Since establishing an accurate age of drinking onset is critical, trained clinical nurses and psychologists from the NSW BTRC performed extensive interviews with the human volunteers and their families. Age of drinking onset and alcohol drinking history were derived from personal interviews with the volunteers as well as medical records and next‐of‐kin interviews. In cases where the age of drinking onset was unclear, an age of 25 was recorded.^32^ Individuals with AUD reported an average age of drinking onset of 16.6 (±0.5) years of age, which was compared with age‐matched CONs whose average age of drinking onset was 24.5 (±0.5) years of age. It is interesting that 100% of individuals with AUD were able to recall their age of drinking onset, whereas only 10% of CONs (i.e., one individual) were able to provide accurate information. Only individuals with AUD uncomplicated by liver cirrhosis and/or nutritional deficiencies were included in the present study. All psychiatric and AUD diagnoses were confirmed using the Diagnostic Instrument for Brain Studies that complies with the Diagnostic and Statistical Manual of Mental Disorders.^33^

**TABLE 1:** Demographics of human male post‐mortem moderate drinking control (CON) and alcohol use disorder (AUD) individuals

Classification	Age of death	Brain weight (g)	PMI (h)	Brain pH	RIN	Clinical cause of death	Age of drinking onset	Lifetime alcohol consumption (kg)	Years drinking	BMI (kg/m^2^)
CON	53	1590	16	6.8	7.9	Cardiac	25	102	28	26
CON	48	1330	24	6.7	6.9	Cardiac	25	17	23	24
CON	44	1220	50	6.6	7.1	Cardiac	25	28	19	28
CON	60	1420	28	6.8	8	Cardiac	25	*unknown*	35	29
CON	46	1320	29	6.1	4.4	Cardiac	25	115	21	*unknown*
CON	24	1490	43	6.3	6.2	Cardiac	20	15	4	38
CON	50	1426	30	6.4	7.5	Cardiac	25	*unknown*	*unknown*	28
CON	62	1430	46	7	8.8	Cardiac	25	5	*7*	*33*
CON	50	1596	40	6.9	8.6	Cardiac	25	18	25	29
CON	40	1441	27	6.8	7.4	Cardiac	25	47	16	35
AUD	25	1400	43.5	6.7	6.9	Toxicity	16	552	9	19
AUD	50	1520	17	6.3	7	Cardiac	18	2453	32	24
AUD	44	1360	15	6.5	7.9	Cardiac	20	639	10	24
AUD	42	1400	41	6.5	8	Toxicity	18	1472	24	24
AUD	45	1580	18.5	6.6	7.9	Respiratory	15	1800	29	29
AUD	61	1588	59	6.6	6.1	Cardiac	16	8052	43	25
AUD	49	1600	44	6.4	6.4	Cardiac	16	1012	33	26
AUD	49	1420	16	6.2	6.2	Cardiac	14	613	35	33
AUD	61	1340	23.5	6.9	8.3	Cardiac	17	5621	44	25
AUD	50	1470	34.5	6.9	7.3	Respiratory	16	5212	34	28

Abbreviations: Age of drinking onset is significantly different, *t*(18) = − 10.7, *p* = 0.0001, between CON (24.5 ± 0.5) and AUD individuals (16.6 ± 0.5). Lifetime alcohol consumption, CON: 43 kg ± 15, AUD: 2743 kg ± 829; *t*(9.0) = 3.3, *p* = 0.010, Welch's *t* test, and body mass index (BMI), CON: 30 kg/m^2^ ± 1, AUD: 26 kg/m^2^ ± 1; *t*(17) = − 2.3, *p* = 0.035, are significantly different between groups. No differences were observed regarding age of death (*p* = 0.983), brain weight (*p* = 0.401), post‐mortem interval (PMI; *p* = 0.728), brain pH (*p* = 0.502), RNA integrity number (RIN; *p* = 0.869), or years drinking (*p* = 0.077).

### 2.2 RNA extraction and reverse transcription PCR (RTPCR)

Total RNA was extracted from frozen human CON and AUD OFC tissue samples by homogenization in TRI reagent (Sigma‐Aldrich, St. Louis, MO) following the single‐step method of RNA isolation.^34^ RNA quality and concentration was determined using NanoDrop 1000 (ThermoFisher Scientific, Waltham, MA). Total RNA was reverse transcribed as previously described.^3^ RTPCR was run on a Bio‐Rad CFX system (Bio‐Rad Laboratories, Hercules, CA) using SYBER Green PCR Master Mix (Life Technologies, Carlsbad, CA). RTPCR was run with an initial activation for 10 min at 95°C, followed by 40 cycles of denaturation (95°C, 15 s), annealing/extension (57–58°C, 1 min), and melt curve. The primer sequences are presented in Table 2. Differences in primer expression between groups are expressed as cycle time (Ct) values normalized with β‐actin, and relative differences between groups calculated and expressed as the percent difference relative to CONs.

**TABLE 2:** Primers for human reverse transcription PCR

Primer	Forward	Reverse
*NOX1*	5′–3′	5′–3′
*NOX2*	5′‐CCA TCC GGA GGT CTT ACT TTG A‐3′	5′‐ACG TAC AAT TCG TTC AGC TCC AT‐3′
*NOX3*	5′‐ACC GTG GAG GAG GCA ATT AGA CAA‐3′	5′‐TTC CAG GTT GAA GAA ATG CGC CAC‐3′
*NOX4*	5′‐CTT CCG TTG GTT TGC AGA TT‐3′	5′‐TGA ATT GGG CCA CAA CAG A‐3′
*NOX5*	5′‐TGC TGC TCC TCC TCA TGT TCA TCT‐3′	5′‐TCC AGA AGT TGG GCC CAT GAA AGA‐3′
*DUOX1*	5′‐AAC AAT TTG TGC GGC TAC GGG ATG‐3′	5′‐TCC TGC AGG GTG GTA TTT CGG ATT‐3′
*DUOX2*	5′‐AGT ACA AGC GCT TCG TGG AGA ACT‐3′	5′‐TCT GCA AAC ACG CCA ACA CAG ATG‐3′
*ATF4*	5′‐CCA TTT CTA CTT TGC CCG CC‐3′	5′‐GGC GCT CGT TAA ATC GCT TC‐3′
*ATF6*	5′‐ACG GAG TAT TTT GTC CGC CT‐3′	5′‐GGC TCC CCC ATT TCA CAA GT‐3′
*CHOP*	5′‐ACG GGA CCT ACT ACG AGT GT‐3′	5′‐GCT TCA CGG CAA AGA GAT CG‐3′
*EIF2α*	5′‐CGA CAA CCC TGG AGA GAA CA‐3′	5′‐TGC CTC GCA AGT TCA GTC TC‐3′
*GRP78*	5′‐CAG AAT CGC CTG ACA CCT GA‐3′	5′‐GGG CCT GCA CTT CCA TAG AG‐3′
*PERK*	5′‐AAA GTC CAC CTT CCC CAA CAA‐3′	5′‐CCA CCT GAG TGA CAG CCT A‐3′
*XBP1*	5′‐GCC TTG TAG TTG AGA ACC AGG A‐3′	5′‐ACG TAG TCT GAG TGC TGC GG‐3′
*CASP3*	5′‐CCC AGG CCG TGA GGA GTT A‐3′	5′‐TTA ATG AGA ATG GGG GAA GAG GC‐3′
*CASP7*	5′‐TTT GTA GAG CGA GGG GCC AA‐3′	5′‐TGG AAG AGC CCA AAG CGA C‐3′
*CASP8*	5′‐AGG CAC AGA GAT TAA GTC CAT T‐3′	5′‐GAC ACA CAG ACT CGA ATG CC‐3′
*CASP9*	5′‐GGC CCT GGA TGT TAG GAT GGA T‐3′	5′‐GAC TTG GAA AGG TCA CAG AAG G‐3′
*CASP12*	5′‐TTG ACC TTT TGG GGA TGC GA‐3′	5′‐GCT TGG TCC CAC AGA TTC CA‐3′
*β‐Actin*	5′‐GCA TGG GTC AGA AGG ATT CCT‐3′	5′‐TCG TCC CAG TTG GTG ACG AT‐3′

### 2.3 Immunohistochemistry

Paraffin‐embedded post‐mortem human OFC sections were deparaffinized, washed in PBS, and antigen retrieval performed by incubation in Citra solution (BioGenex, San Ramon, CA) for 1 h at 70°C. Following incubation in blocking solution (MP Biomedicals, Solon, OH), slides were incubated in a primary antibody solution for 24 h at 4°C. Primary antibodies, dilutions, and validation information are included in Table 3. Slides were incubated for 1 h in biotinylated secondary antibody (1:200; Vector Laboratories, Burlingame, CA) and then for 1 h in avidin–biotin complex solution (Vector Laboratories). The chromogen nickel‐enhanced diaminobenzidine (Sigma‐Aldrich) was used to visualize immunoreactivity. Slides were dehydrated and cover slipped. Negative control for non‐specific binding was conducted employing the above‐mentioned procedures with omission of the antibody.

**TABLE 3:** Primary antibodies used for immunohistochemistry in the post‐mortem human orbitofrontal cortex

Antibody	Isotype (IgG)	Source/purification	Dilution	Company, catalogue number	Validation
GRP78	Rabbit	Polyclonal	1:100	Abcam Inc., #ab21685	WB (Abcam)
PERK	Rabbit	Polyclonal	1:100	Novus Biologicals, #NBP1‐80930	IHC (Novus Biologicals)
pIRE1 (phospho S724)	Rabbit	Polyclonal	1:100	Abcam Inc., #ab48187	WB (Abcam)
ATF6	Rabbit	Polyclonal	1:150	Abcam Inc., #ab203119	WB (Abcam)
CHOP	Rabbit	Polyclonal	1:00	Abcam Inc., #ab10444	WB, IHC^a^
NOX2 (gp91‐phox)	Mouse	Monoclonal	1:50	Santa Cruz Biotechnology, #sc‐130,543	WB (Santa Cruz)
Cleaved Caspase 3	Rabbit	Polyclonal	1:100	Cell Signaling Technology, #9661	WB, IHC‐Block (Cell Signaling)
Caspase 12	Rabbit	Polyclonal	1:100	Invitrogen, #PA5‐20034	WB, IHC (Invitrogen)
4‐HNE	Rabbit	Polyclonal	1:100	Abcam Inc., #ab46545	WB (Abcam)
8‐OHdG	Mouse	Monoclonal	1:50	Santa Cruz Biotechnology, #sc‐66,036	IHC^b^
NeuN	Chicken	Polyclonal	1:100	Novus Biologicals, #NBP2‐10491	IHC (Novus Biologicals)
MAP‐2	Rabbit	Monoclonal	1:150	Abcam Inc., #ab183830	Flow cytometry, IHC (Abcam)

*Note*: Validation information includes references as well as methods used for validation and source.

^a^ Ren et al.^35^

^b^ Lin et al.^36^

### 2.4 Microscopic quantification

BioQuant Nova Advanced Image Analysis software (R&M Biometric, Nashville, TN) was used for image capture and quantification of immunohistochemistry. Representative images were captured using an Olympus BX50 microscope and Sony DXC‐390 video camera linked to a computer. For each measure, the microscope, camera, and software were background corrected and normalized to preset light levels to ensure fidelity of data acquisition. A modified unbiased stereological quantification method was used to quantify GRP78‐, PERK‐, pIRE1‐, ATF6‐, CHOP‐, cleaved caspase 12‐, 4‐HNE‐, 8‐OHdG‐, NeuN‐, and MAP 2‐immunoreactive (+IR) cells in the post‐mortem human OFC. We previously reported that comparison of traditional unbiased stereological methodology with our modified unbiased stereological approach yielded nearly identical values relative to control subjects.^37^ The outlined regions of interest were determined and data expressed as cells/mm^2^.

### 2.5 Fluorescent immunohistochemistry and microscopy

Paraffin‐embedded human OFC sections were deparaffinized, washed in PBS, and antigen retrieval performed by incubation in Citra solution (BioGenex) for 1 h at 70°C. For double immunofluorescent immunohistochemistry, slides were incubated in blocking solution (MP Biomedicals) and incubated for 24 h at 4°C in a primary antibody solution consisting of blocking solution with NOX2 in combination with an antibody against either GRP78, ATF6, CHOP, cleaved caspase 3, or caspase 12 (Table 3). For triple immunofluorescent immunohistochemistry, slides were incubated for 24 h at 4°C in a primary antibody solution consisting of blocking solution with cleaved caspase 3 and NeuN in combination with an antibody against either NOX2, GRP78, or ATF6. Slides were washed in PBS and incubated for 1 h at room temperature with the appropriate secondary antibodies (1:1000). Immunoblotting was visualized using Alexa Fluor 350, 488 or 594 dye as necessary. Secondary‐only negative controls were performed without primary antibody incubation. Slides were coverslipped using Prolong Gold Anti‐fade mounting media (Life Technologies). Immunofluorescent images were obtained using a Nikon DS‐Ri2 scope (Nikon Inc., Melville, NY) and colocalization quantified using NIS Elements AR46 analysis software (Nikon Inc.).

### 2.6 Statistical analysis

Statistical analysis was performed using SPSS (Chicago, IL) and GraphPad Prism 8 (San Diego, CA). Sample size determinations were based on previously published studies (Crews et al, 2013; Liu et al, 2020; Vetreno et al, 2021). Two‐tailed Student's *t* tests were used to assess human demographics, RTPCR, and immunohistochemistry data unless otherwise reported. Levene's test for equality of variances was performed for each analysis. When reported in the Results, Welch's *t* tests were used to assess data with unequal variances. Two‐tailed Pearson's *r* were used for all correlative analyses. All values are reported as mean ± SEM, and significance was defined as *p* ≤ 0.05.

## 3 RESULTS

### 3.1 Increased expression of NADPH oxidase genes and oxidative stress markers in the post‐mortem OFC of individuals with AUD

**FIGURE 1:**
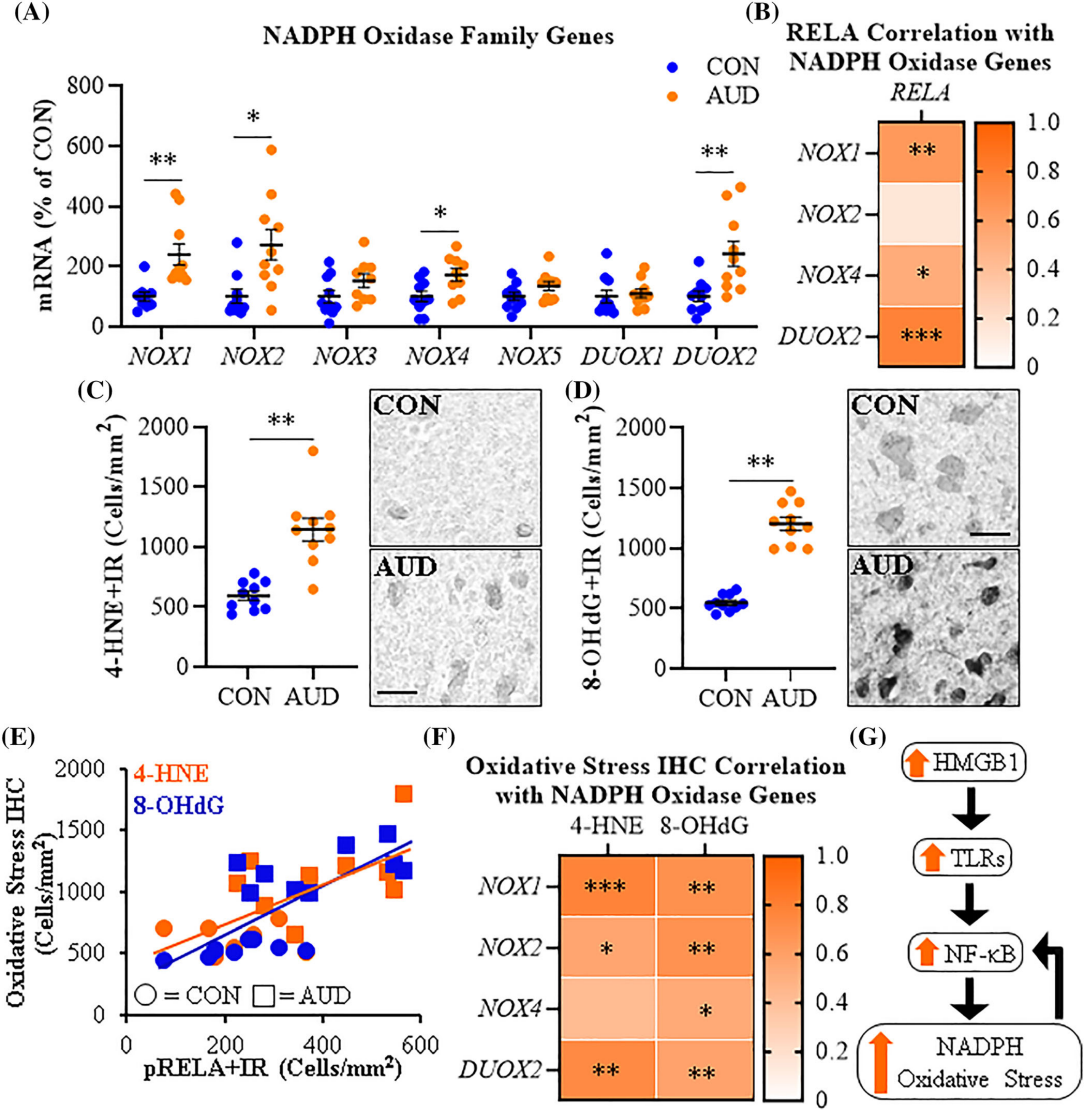
Expression of NADPH oxidase genes and oxidative stress markers in age‐matched moderate drinking control (CON) and alcohol use disorder (AUD) post‐mortem human orbitofrontal cortex (OFC). (A) Reverse transcription PCR (RTPCR) analysis revealed an AUD increase of NOX1 (2.4‐fold), NOX2 (2.7‐fold), NOX4 (1.7‐fold), and DUOX2 (2.4‐fold) in the OFC relative to CONs. (B) Heat map depicting positive Pearson's *r* correlation of the proinflammatory nuclear transcription NF‐κB factor gene RELA68 with the induced NADPH oxidase genes NOX1 (*r* = 0.64, *p* = 0.003), NOX4 (*r* = 0.66, *p* = 0.002), and DUOX2 (*r* = 0.78, *p* = 0.00004) in the post‐mortem human OFC. NOX2 did not correlate with expression of RELA. Darker orange colour indicates higher Pearson's *r* value (e.g., DUOX2) while lighter orange‐white indicates lower Pearson's *r* value (e.g., NOX2). (C) Modified unbiased stereological assessment revealed an approximate 2.0‐fold increase of 4‐hydroxynonenal (4‐HNE) in the AUD OFC relative to CONs. Representative photomicrographs of 4‐HNE + IR in the OFC of a CON and AUD individual. Scale bar = 30 μm. (D) Modified unbiased stereological assessment revealed an approximate 2.2‐fold increase of 8‐hydroxy‐2′‐deoxyguanosine (8‐OHdG) in the AUD OFC relative to CONs. Representative photomicrographs of 8‐OHdG + IR in the OFC of a CON and AUD individual. Scale bar = 30 μm. (E) Across individuals, immunohistological expression of phosphorylated (activated) NF‐κB p65 (pRELA)68 positively correlates with expression of 4‐HNE + IR (orange; *r* = 0.66, *p* = 0.003) and 8‐OHdG + IR (blue; *r* = 0.73, *p* = 0.0006) in the post‐mortem human OFC. (F) Heat map with high Pearson's *r* values (orange) depicting positive correlations of the oxidative stress markers 4‐HNE (NOX1: *r* = 0.77, *p* = 0.0001; NOX2: *r* = 0.56, *p* = 0.011; NOX4: *r* = 0.42, *p* = 0.072; DUOX2: *r* = 0.73, *p* = 0.003) and 8‐OHdG (NOX1: *r* = 0.69, *p* = 0.001; NOX2: *r* = 0.68, *p* = 0.009; NOX4: *r* = 0.53, *p* = 0.021; DUOX2: *r* = 0.58, *p* = 0.007) with the induced NADPH oxidase genes in the post‐mortem human OFC. (G) Simplified schematic depicting AUD‐induced high‐mobility group box 1 (HMGB1) signalling through Toll‐like receptors (TLRs) to NF‐κB resulting in induction of NADPH oxidases and oxidative stress. Previous studies from our laboratory reported upregulation of proinflammatory HMGB1‐TLR‐NFκB neuroimmune signalling cascades leads to neurodegeneration in the post‐mortem human OFC of individuals with AUD68. RTPCR analyses were run in triplicate. Data are presented as mean ± SEM. *p ≤ 0.05, **p < 0.01, ***p < 0.001

Reactive oxygen species (ROS) are an extensively studied mechanism of neuronal degeneration associated with neurodegenerative diseases. In preclinical studies, we reported that ethanol exposure and proinflammatory signalling increase brain levels of the NADPH oxidase gp91^phox^ (NOX2) and ROS in association with cell death.^1^ NADPH oxidase is a multicomponent membrane‐bound enzyme complex that catalyses the production of superoxide (O_2_^−^) and other ROS. This enzyme family consists of five NADPH oxidases (NOX1‐NOX5) and two dual oxidases (DUOX1 and DUOX2). With the exception of NOX2, expression profiles of NOXs and DUOXs in human AUD have not been clearly defined, prompting assessment of NADPH oxidase expression in the post‐mortem human OFC of individuals with AUD (*n* = 10) relative to age‐matched moderate drinking CONs (*n* = 10). In this cohort, individuals with AUD averaged 2743 kg of lifetime alcohol consumption whereas CONs averaged 43 kg of lifetime consumption across their lifetime. Post‐mortem brain weight (CON: 1426 g; AUD 1468 g), post‐mortem interval (CON: 33 h; AUD: 31 h), and RNA integrity number (CON: 7.3; AUD: 7.2) did not differ significantly between CON and AUD individuals (Table 1). Determination of NADPH oxidase mRNA gene expression revealed an AUD‐associated increase of *NOX1* (2.4‐fold), *t*(11.9) = 3.7, *p* = 0.003, Welch's *t* test, *NOX2* (2.7‐fold), *t*(12.6) = 3.1, *p* = 0.008, Welch's *t* test, and *NOX4* (1.7‐fold), *t*(17) = 2.6, *p* = 0.017, in the OFC relative to CONs. *NOX3* and *NOX5* levels were unchanged in this cohort. Expression of *DUOX2* mRNA increased 2.4‐fold, *t*(12.1) = 3.2, *p* = 0.008, Welch's *t* test, in the AUD OFC relative to CONs (Figure 1A) whereas *DUOX1* expression did not differ between groups. Together, these data reveal increased expression of several genes within the NADPH oxidase family in the post‐mortem human OFC of individuals with AUD.

**FIGURE 2:**
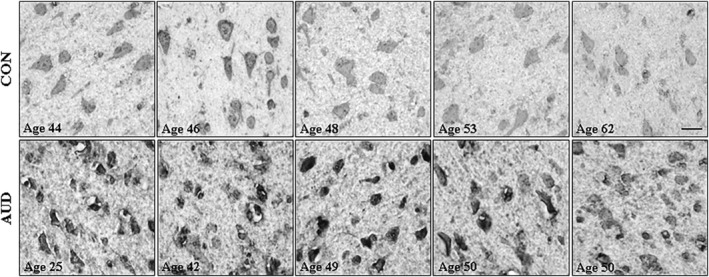
Immunohistological expression of the DNA oxidation marker 8‐hydroxy‐2′‐deoxyguanosine (8‐OHdG) in age‐matched moderate drinking control (CON) and alcohol use disorder (AUD) post‐mortem human orbitofrontal cortex (OFC). Representative photomicrographs depicting the extent of 8‐OHdG immunoreactivity in the OFC of individuals with AUD relative to moderate drinking CONs. Scale bar = 30 μm

Preclinical models of chronic ethanol exposure link activation of the proinflammatory nuclear transcription factor NF‐κB to generation of proinflammatory oxidases and ROS.^1^ In a previous study, we reported a link between increased expression of Toll‐like receptors (TLRs) and the cytokine‐like endogenous TLR agonist high‐mobility group box 1 (HMGB1) with downstream activation of NF‐κB p65 (RELA) in the post‐mortem OFC.^3^ These same individuals were used in the current study, allowing correlations of NADPH oxidase expression with RELA. We report that *RELA* mRNA expression levels positively correlate with *NOX1* (*r* = 0.64, *p* = 0.003), *NOX4* (*r* = 0.52, *p* = 0.023), and *DUOX2* (*r* = 0.78, *p* < 0.001) (Figure 1B). Although correlation does not establish causal relationships or directionality, these correlations across individuals are consistent with an AUD‐induced association of NF‐κB signalling and induction of NADPH oxidases in the post‐mortem OFC. Immunohistochemical determination of oxidative stress markers in the OFC revealed an AUD‐induced approximate two‐fold increase of the lipid peroxidation marker 4‐hydroxynonenal (4‐HNE), *t*(18) = 5.5, *p* < 0.001 (Figure 1C), and an approximate 2.2‐fold increase of the DNA oxidation marker 8‐hydroxy‐2′‐deoxyguanosine (8‐OHdG), *t*(11.6) = 11.2, *p* < 0.001, Welch's *t* test (Figures 1D and 2), relative to CONs. Photomicrographs of these oxidative stress‐associated proteins show clear increases in the AUD OFC relative to moderate drinking CONs. Expression of phosphorylated (activated) RELA + IR positively correlates with both 4‐HNE (*r* = 0.66, *p* = 0.003) and 8‐OHdG (*r* = 0.73, *p* < 0.001) supporting NF‐κB activation as contributing to AUD‐induced oxidative stress in the OFC (Figure 1E). Further, we report that expression of the lipid peroxidation marker 4‐HNE and the DNA oxidation marker 8‐OHdG positively correlate with induced NADPH oxidase family genes, consistent with concerted activation of oxidative stress in the AUD OFC (Figure 1F). These findings are consistent with induction of NADPH oxidase‐mediated oxidative stress in the OFC of individuals with AUD through a mechanism involving NF‐κB activation (Figure 1G).

### 3.2 Increased expression of ER stress markers in the post‐mortem OFC of individuals with AUD

**FIGURE 3:**
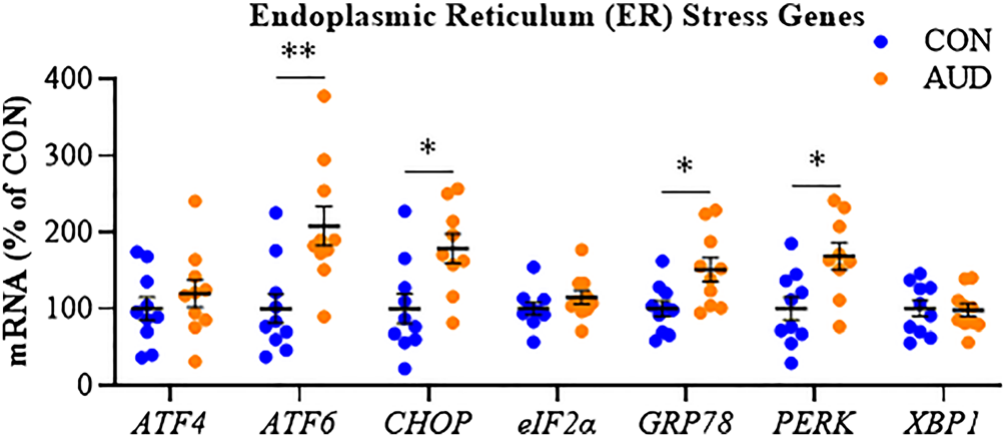
Expression of endoplasmic reticulum (ER) stress genes in age‐matched moderate drinking control (CON) and alcohol use disorder (AUD) post‐mortem human orbitofrontal cortex (OFC). Reverse transcription PCR (RTPCR) analysis revealed an AUD increase of ATF6 (2.1‐fold), CHOP (1.8‐fold), GRP78 (1.5‐fold), and PERK (1.7‐fold) in the OFC relative to CONs. RTPCR analyses were run in triplicate. Data are presented as mean ± SEM. *p ≤ 0.05, **p < 0.01

**FIGURE 4:**
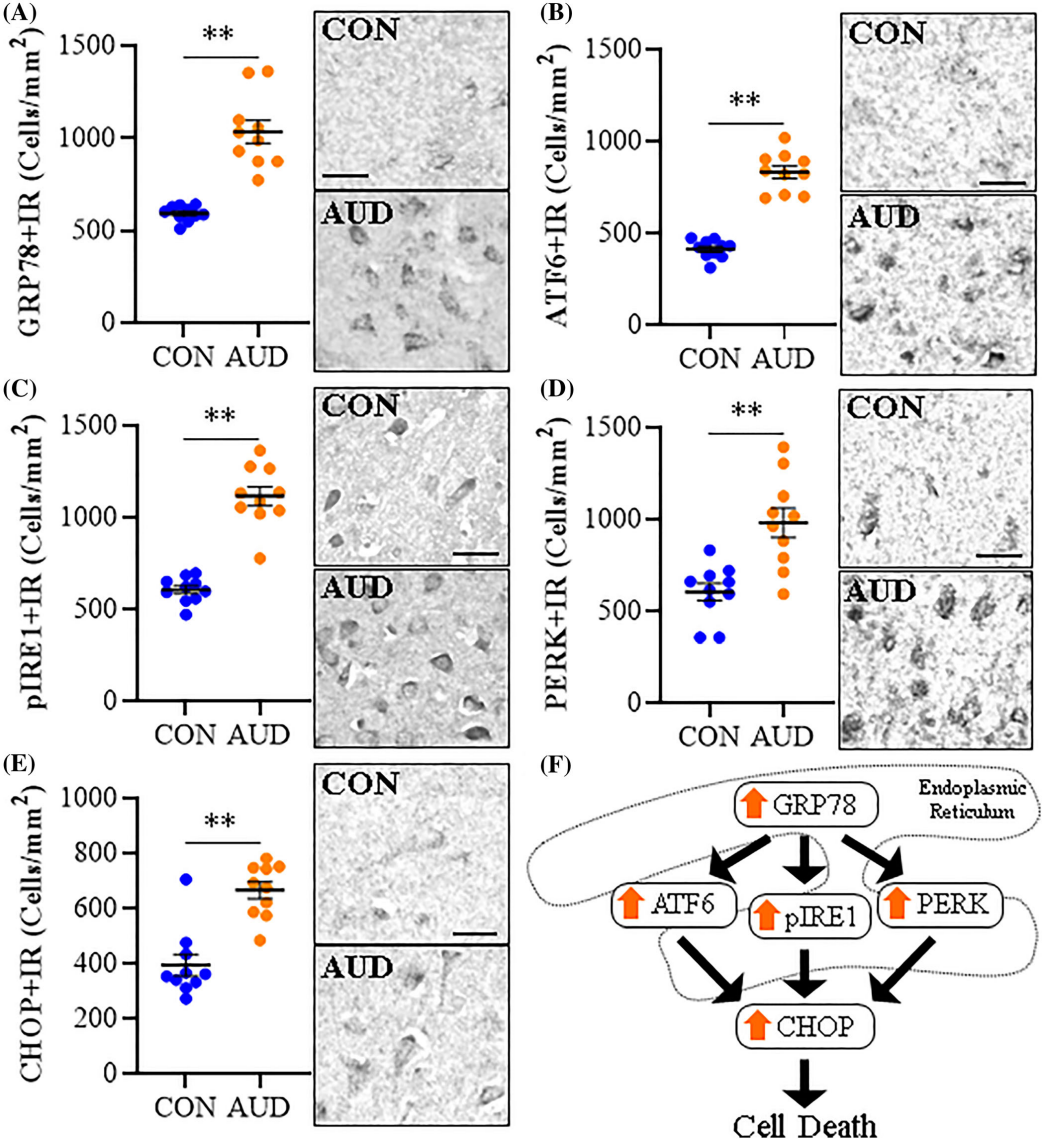
Immunohistological expression of endoplasmic reticulum (ER) stress proteins in age‐matched moderate drinking control (CON) and alcohol use disorder (AUD) post‐mortem human orbitofrontal cortex (OFC). (A) Modified unbiased stereological assessment revealed an approximate 1.7‐fold increase of glucose‐regulated protein 78 (GRP78) in the AUD OFC relative to CONs. Representative photomicrographs of GRP78 + IR in the OFC of a CON and AUD individual. Scale bar = 30 μm. (B) Modified unbiased stereological assessment revealed an approximate 2.0‐fold increase of activating transcription factor 6 (ATF6) in the AUD OFC relative to CONs. Representative photomicrographs of ATF6 + IR in the OFC of a CON and AUD individual. Scale bar = 30 μm. (C) Modified unbiased stereological assessment revealed an approximate 1.8‐ fold increase of phosphorylated inositol requiring protein 1 (pIRE1) in the AUD OFC relative to CONs. Representative photomicrographs of pIRE1 + IR in the OFC of a CON and AUD individual. Scale bar = 30 μm. (D) Modified unbiased stereological assessment revealed an approximate 1.6‐fold increase of protein kinase RNA‐like endoplasmic reticulum kinase (PERK) in the AUD OFC relative to CONs. Representative photomicrographs of PERK + IR in the OFC of a CON and AUD individual. Scale bar = 30 μm. (E) Modified unbiased stereological assessment revealed an approximate 1.7‐fold increase of C/EBP homologous protein (CHOP) in the AUD OFC relative to CONs. Representative photomicrographs of CHOP + IR in the OFC of a CON and AUD individual. Scale bar = 30 μm. (F) Simplified schematic depicting activation of the ER stress cascade in the OFC of individuals with AUD. Data are presented as mean ± SEM. *p ≤ 0.05, **p < 0.01

ROS‐producing NADPH oxidases contribute to endoplasmic reticulum (ER) stress.^17^ ER stress occurs as a consequence of unfolded and misfolded protein accumulation within the ER that triggers the unfolded protein response (UPR). The UPR involves dissociation of glucose‐regulated protein 78 (GRP78) from ER resident transmembrane sensor proteins, including inositol‐requiring kinase/endonuclease 1α (IRE1α), protein kinase RNA‐like endoplasmic reticulum kinase (PERK), and activating transcription factor 6 (ATF6), which signal through the pro‐apoptotic transcription factor C/EBP homologous protein (CHOP).^27^ Assessment of ER stress‐associated genes revealed an AUD‐induced increase of *GRP78* (1.5‐fold), *t*(18) = 2.8, *p* = 0.013, *ATF6* (2.1‐fold), *t*(18) = 3.4, *p* = 0.003, *PERK* (1.7‐fold), *t*(16.2) = 2.9, *p* = 0.010, Welch's *t* test, and *CHOP* (1.7‐fold), *t*(16.9) = 2.9, *p* = 0.010, in the OFC relative to CONs (Figure 3). Gene expression of *ATF4*, *eIF2a*, and *XBP1* were unchanged between groups. Immunohistological determination of ER stress‐associated proteins revealed an AUD‐induced increase of GRP78 + IR (1.7‐fold), *t*(9.8) = 7.0, *p* < 0.001, Welch's *t* test (Figure 4A), ATF6 + IR (2.0‐fold), *t*(18) = 11.1, *p* < 0.001 (Figure 4B), pIRE1 + IR (1.8‐fold), *t*(18) = 9.0, *p* < 0.001 (Figure 4C), PERK + IR (1.6‐fold), *t*(18) = 4.1, *p* < 0.001 (Figure 4D), and CHOP + IR (1.7‐fold), *t*(18) = 5.5, *p* < 0.001 (Figure 4E) relative to CONs. Photomicrographs of these ER stress‐associated proteins show clear increases in the AUD OFC relative to moderate drinking CONs. Thus, these data reveal increased expression of the ER chaperone GRP78, the downstream transmembrane proteins PERK, pIRE1, and ATF6, and the pro‐apoptotic transcription factor CHOP, consistent with activation of the ER stress and the UPR cascade in the OFC of individuals with AUD (Figure 4F).

**FIGURE 5:**
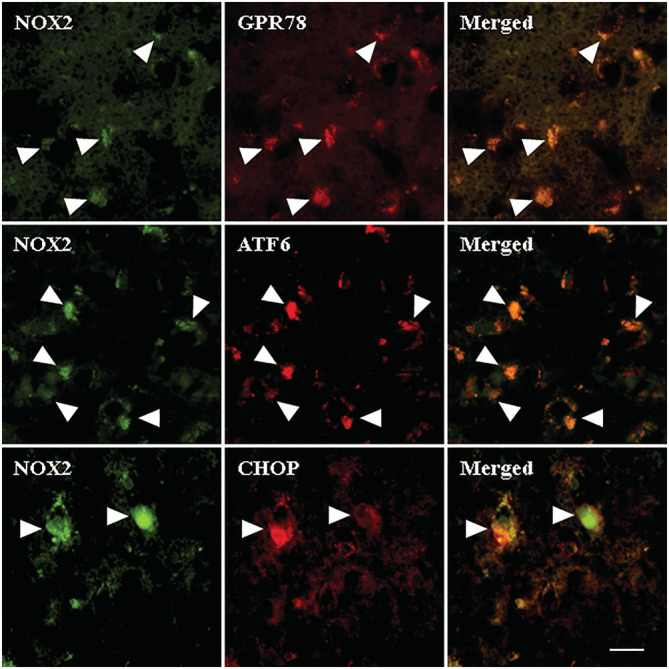
Fluorescent immunohistochemical photomicrographs of the NADPH oxidase NOX2 colocalization with endoplasmic reticulum (ER) stress markers in the post‐mortem human orbitofrontal cortex (OFC). (TOP) Photomicrographs of NOX2 (green) colocalization with the ER stress marker glucose‐regulated protein 78 (GRP78; red) in the post‐mortem human OFC of an individual with alcohol use disorder (AUD). White arrows indicate NOX2 + IR cells that colocalize with GRP78 (yellow). (MIDDLE) Photomicrographs of NOX2 (green) colocalization with the ER stress marker activating transcription factor 6 (ATF6; red) in the postmortem human OFC of an individual with AUD. White arrows indicate NOX2 + IR cells that colocalize with ATF6 (yellow). (BOTTOM) Photomicrographs of NOX2 (green) colocalization with the ER stress marker C/EBP homologous protein (CHOP; red) in the post‐mortem human OFC of an individual with AUD. White arrows indicate NOX2 + IR cells that colocalize with CHOP (yellow). These findings suggest that formation of reactive oxygen species from NADPH oxidases (NOX2) contributes to ER stress induction within the same cells. Scale bar = 20 μm

Since NADPH oxidase and ER stress‐associated markers increase in the OFC of individuals with AUD consistent with an interaction of ROS and ER stress within cells, we assessed colocalization of NOX2 with the ER stress markers GPR78, ATF6, and CHOP. Co‐localization of NOX2 with ER stress proteins suggests that oxidative stress and ROS generation from NADPH oxidases contribute to ER stress within the same cells (Figure 5). Neuroinflammation and oxidative stress are linked to ER stress in several neurodegenerative disorders,^38^ and we report that induced NADPH oxidase family genes are positively correlated with the majority of induced ER stress‐associated genes (Table 4). For example, *DUOX2* shows robust positive correlations with *ATF6* (*r* = 0.87, *p* = 0.0000006), *PERK* (*r* = 0.86, *p* = 0.0000009), and *CHOP* (*r* = 0.88, *p* = 0.0000003). Similarly, expression of the oxidative stress lipid peroxidation marker 4‐HNE and the DNA oxidation marker 8‐OHdG both positively correlate with expression of ER stress‐associated proteins (Table 5). Taken together, these data suggest that induction of NADPH oxidative stress is associated with ER stress within the same cell in the OFC of individuals with AUD.

**TABLE 4:** Correlation of NADPH oxidative family genes with endoplasmic reticulum (ER) stress genes in the post‐mortem human orbitofrontal cortex (OFC) of age‐matched moderate drinking control (CON) and alcohol use disorder (AUD) individuals

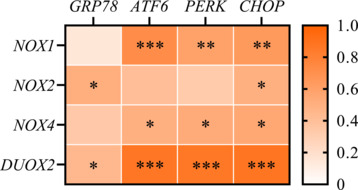

*Note*: Pearson's *r* correlations assessed the association of *NOX*/*DUOX* genes with ER stress genes in post‐mortem human OFC tissue samples from CON and AUD individuals. Pearson's *r* correlation coefficients were used with two‐tailed significance. Darker orange colour indicates higher Pearson's *r* value while lighter orange‐white indicates lower Pearson's *r* value.

* *p* < 0.05.

** *p* < 0.01.

*** *p* < 0.001.

**TABLE 5:** Correlation of oxidative stress immunohistochemistry (IHC) markers with endoplasmic reticulum (ER) stress IHC markers in the post‐mortem human orbitofrontal cortex (OFC) of age‐matched moderate drinking control (CON) and alcohol use disorder (AUD) individuals

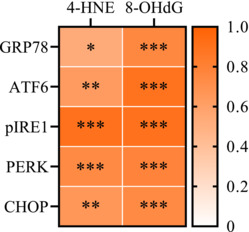

*Note*: Pearson's *r* correlations assessed the association of 4‐HNE and 8‐OHdG oxidative stress markers with ER stress markers in post‐mortem human OFC tissue samples from CON and AUD individuals. Pearson's *r* correlation coefficients were used with two‐tailed significance. Darker orange colour indicates higher Pearson's *r* value while lighter orange‐white indicates lower Pearson's *r* value.

* *p* < 0.05.

** *p* < 0.01.

*** *p* < 0.001.

### 3.3 Increased expression of cell death‐related caspase cascades is associated with NADPH oxidative stress and ER stress in the post‐mortem OFC of individuals with AUD

**FIGURE 6:**
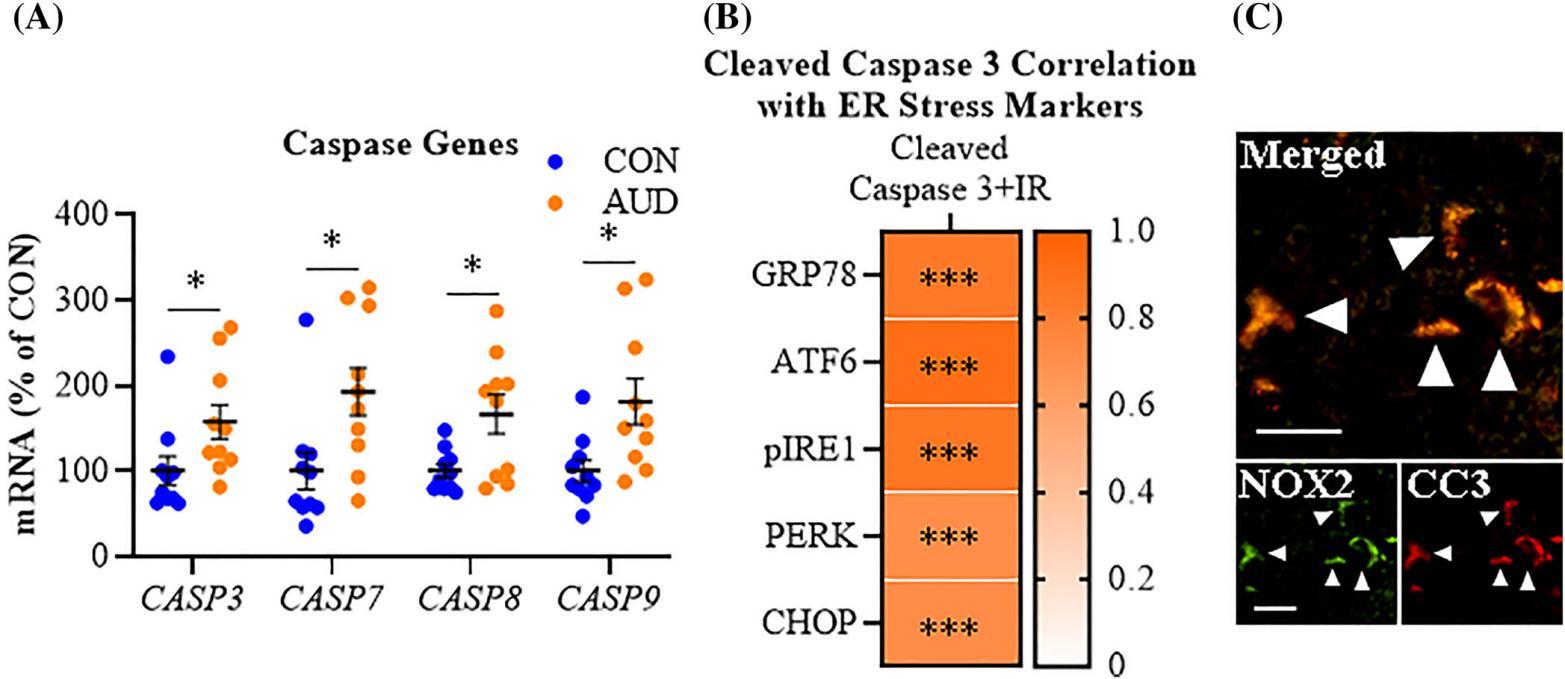
Expression of cell death‐related caspase genes and cleaved caspase 3 are associated with endoplasmic reticulum (ER) stress markers in age‐matched moderate drinking control (CON) and alcohol use disorder (AUD) post‐mortem human orbitofrontal cortex (OFC). (A) Reverse transcription PCR (RTPCR) analysis revealed an AUD increase of CASP3 (1.6‐fold), CASP7 (1.9‐fold), CASP8 (1.7‐fold), and CASP9 (1.8‐fold) in the OFC relative to CONs. (B) Heat map with high Pearson's *r* values (orange) depicting positive correlations of the cell death protease cleaved caspase 3 58 with the ER stress proteins GRP78, ATF6, pIRE1, PERK, and CHOP in the post‐mortem human OFC. (C) Photomicrographs of NOX2 (green) colocalization with the cell death marker cleaved caspase 3 (red) in the post‐mortem human OFC of an individual with AUD. White arrows indicate NOX2 + IR cells that colocalize with cleaved caspase 3 (yellow). Scale bar = 20 μm. RTPCR analyses were run in triplicate. Data are presented as mean ± SEM. *p ≤ 0.05, **p < 0.01, ***p < 0.001

Activation of caspase cascades is implicated in ER stress‐mediated cell death^39^ and preclinical studies report that treatment with the NOX inhibitor diphenyliodonium decreases chronic ethanol‐induced ROS generation and increased expression of the cell death marker cleaved caspase 3.^1^ To confirm and extend these studies, we assessed multiple caspase genes in the OFC of individuals with AUD relative to CONs. We report an AUD‐induced increase of *CASP3* (1.6‐fold), *t*(18) = 2.2, *p* = 0.042, *CASP7* (1.9‐fold), *t*(18) = 2.6, *p* = 0.017, *CASP8* (1.7‐fold), *t*(11.0) = 2.8, *p* = 0.019, Welch's *t* test, and *CASP9* (1.8‐fold), *t*(12.6) = 2.8, *p* = 0.017, Welch's *t* test, in the OFC relative to CONs (Figure 6A). In a previous study,^7^ we reported increased expression of the cell death protease cleaved caspase 3 in the same individuals used in the current study allowing correlations with expression of ER stress‐associated proteins. We report that cleaved caspase 3 + IR is positively correlated with protein expression of GRP78 (*r* = 0.84, *p* = 0.000004), ATF6 (*r* = 0.91, *p* < 0.001), pIRE1 (*r* = 0.86, *p* < 0.001), PERK (*r* = 0.71, *p* < 0.001), and CHOP (*r* = 0.72, *p* < 0.001) (Figure 6B), consistent with AUD inducing ER stress that contributes to cell death in the OFC. We previously reported increased expression of the NADPH oxidase NOX2 in the post‐mortem OFC of individuals with AUD,^1^ and assessment of NOX2 colocalization with the cell death marker cleaved caspase 3 in the present study revealed co‐expression within the same cells (Figure 6C). Thus, these data reveal induction of cell death caspase cascades in association with increases of NADPH oxidative stress and ER stress in the OFC of individuals with AUD.

**FIGURE 7:**
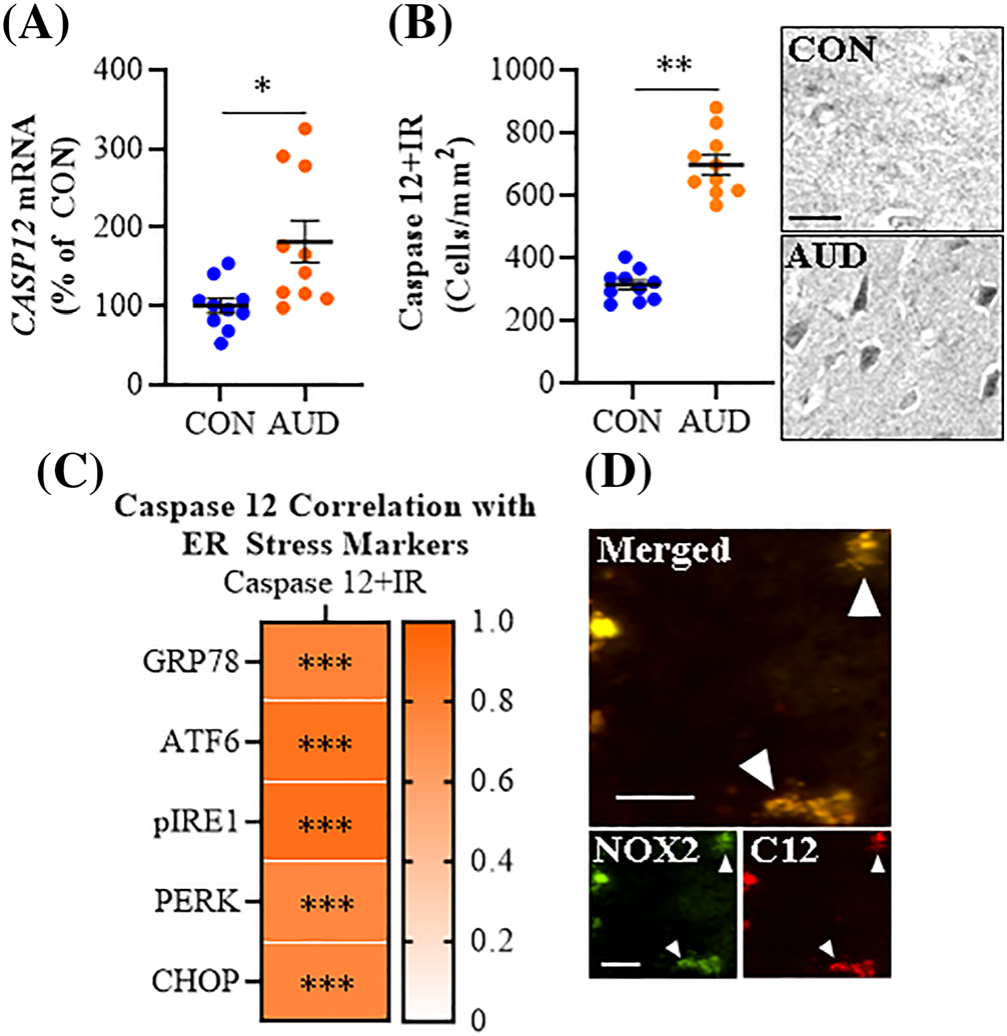
Expression of the endoplasmic reticulum (ER) stress‐associated apoptotic protease caspase 12 in age‐matched moderate drinking control (CON) and alcohol use disorder (AUD) post‐mortem human orbitofrontal cortex (OFC). (A) Reverse transcription PCR (RTPCR) analysis revealed an AUD‐induced 1.8‐fold increase of CASP12 mRNA in the OFC relative to CONs. (B) Modified unbiased stereological assessment revealed an approximate 2.2‐fold increase of caspase 12 in the AUD OFC relative to CONs. Representative photomicrographs of caspase 12 + IR in the OFC of a CON and AUD individual. Scale bar = 30 μm. (C) Heat map with high Pearson's *r* values (orange) depicting positive correlations of the ER apoptotic protease caspase 12 with the ER stress proteins GRP78, ATF6, pIRE1, PERK, and CHOP in the post‐mortem human OFC. (D) Photomicrographs of NOX2 (green) colocalization with caspase 12 (red) in the post‐mortem human OFC of an individual with AUD. White arrows indicate NOX2 + IR cells that colocalize with caspase 12 (yellow). Scale bar = 20 μm. RTPCR analyses were run in triplicate. Data are presented as mean ± SEM. *p ≤ 0.05, **p < 0.01, ***p < 0.001

As caspase 12 is an ER apoptotic protease contributing to ER stress‐associated apoptosis,^40^ we assessed caspase 12 expression in the OFC of individuals with AUD. Determination of *CASP12* gene expression revealed a 1.8‐fold increase in the AUD OFC, *t*(11.3) = 2.9, *p* = 0.015, Welch's *t* test (Figure 7A), relative to CONs. Immunohistochemical assessment of caspase 12 + IR cells revealed a 2.2‐fold increase in the AUD OFC, *t*(13.0) = 10.8, *p* < 0.001, Welch's *t* test (Figure 7B), relative to CONs. Consistent with caspase 12 contributing to ER stress‐induced cell death, caspase 12 + IR positively correlates with expression of GRP78 (*r* = 0.78, *p* < 0.001), ATF6 (*r* = 0.88, *p* < 0.001), pIRE1 (*r* = 0.90, *p* < 0.001), PERK (*r* = 0.75, *p* < 0.001), and CHOP (*r* = 0.76, *p* < 0.001) (Figure 7C). Further, assessment of NOX2 colocalization with caspase 12 revealed co‐expression within the same cells (Figure 7D). Together, these data indicate that chronic alcohol consumption induces NADPH oxidative stress and ER stress that may contribute to increased cell death in the OFC of individuals with AUD.

### 3.4 NADPH oxidative stress and ER stress contribute to neurodegeneration in the post‐mortem OFC of individuals with AUD

**FIGURE 8:**
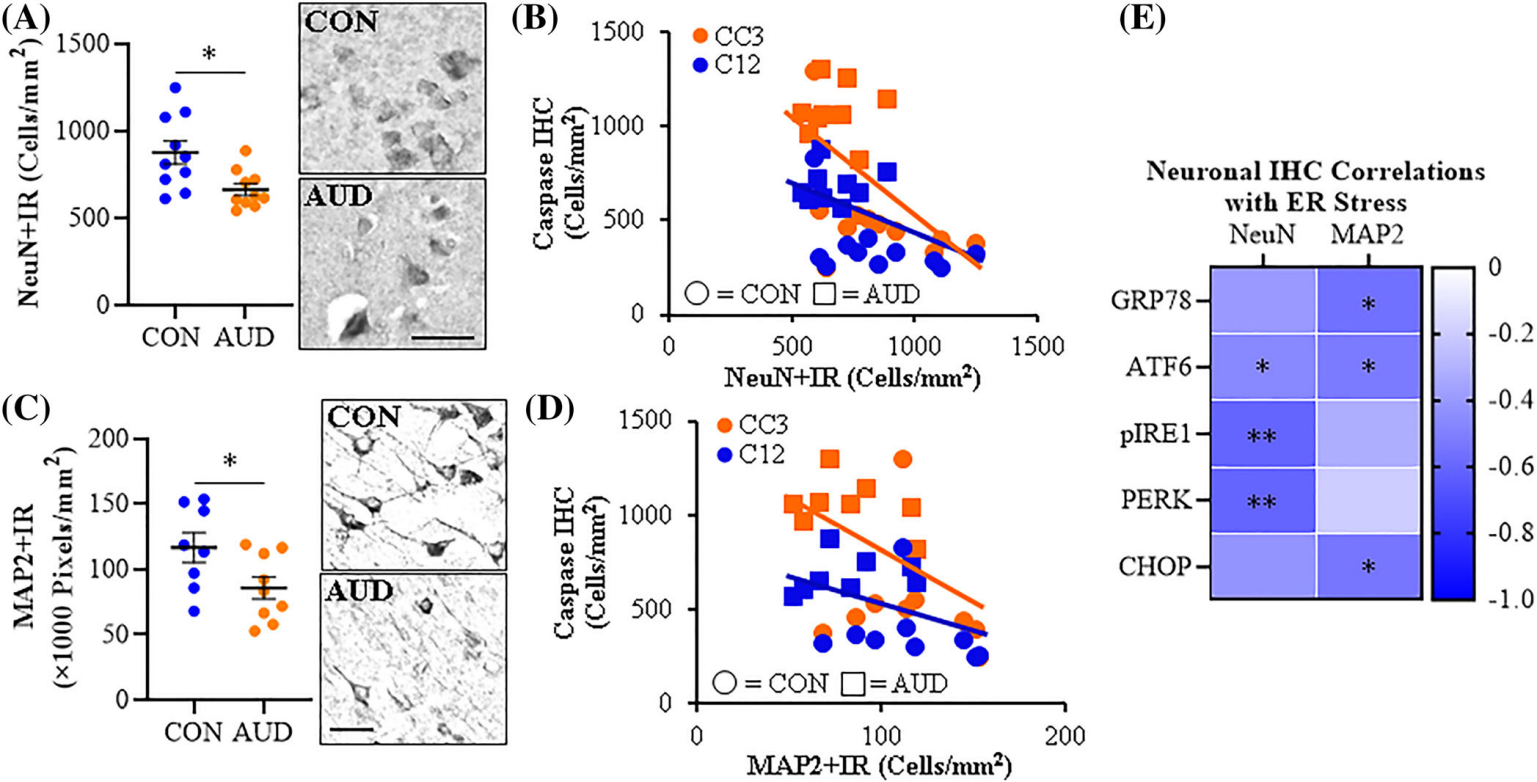
Loss of neurons in the orbitofrontal cortex correlates with expression of cell death and endoplasmic reticulum (ER) stress markers. (A) Modified unbiased stereological assessment revealed an approximate 24% reduction of the neuronal marker NeuN in the AUD OFC relative to CONs. Representative photomicrographs of NeuN + IR in the OFC of a CON and AUD individual. Scale bar = 30 μm. (B) Across individuals, immunohistological expression of NeuN negatively correlates with expression of cleaved caspase 3 and caspase 12 in the post‐mortem human OFC. (C) Modified unbiased stereological assessment revealed an approximate 26% reduction of the neuronal marker MAP2 in the AUD OFC relative to CONs. Representative photomicrographs of MAP2 + IR in the OFC of a CON and AUD individual. Scale bar = 30 μm. (D) Across individuals, immunohistological expression of MAP2 negatively correlates with expression of cleaved caspase 3 but not caspase 12 in the post‐mortem human OFC. (E) Heat map with high Pearson's *r* values (blue) depicting negative correlations of the neuronal markers NeuN and MAP2 with the ER stress proteins GRP78, ATF6, pIRE1, PERK, and CHOP in the post‐mortem human OFC

**FIGURE 9:**
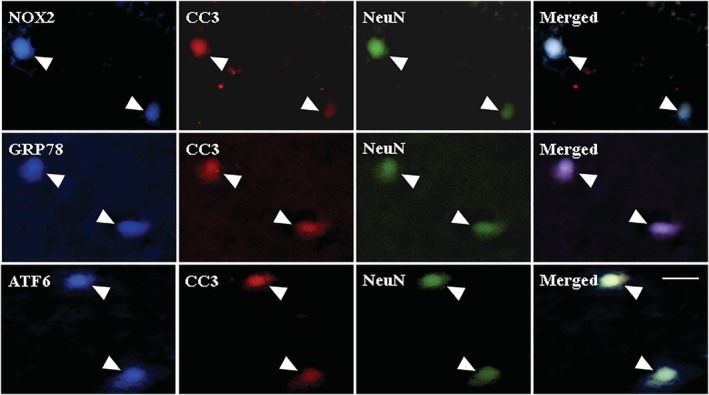
Fluorescent immunohistochemical photomicrographs of cell death marker cleaved caspase 3 and the neuronal marker NeuN colocalization with the NADPH oxidase NOX2 and endoplasmic reticulum (ER) stress markers in the post‐mortem human orbitofrontal cortex (OFC). (TOP) Photomicrographs of NOX2 (blue) and cleaved caspase 3 (CC3; red) colocalization with the neuronal marker NeuN (green) in the postmortem human OFC of individuals with alcohol use disorder (AUD). White arrows indicate NOX2 + IR and CC3 + IR colocalization with NeuN. (MIDDLE) Photomicrographs of the ER stress marker glucose‐regulated protein 78 (GRP78; blue) and CC3 (red) colocalization with the neuronal marker NeuN (green) in the postmortem human OFC of individuals with AUD. White arrows indicate GRP78 + IR and CC3 + IR colocalization with NeuN. (BOTTOM) Photomicrographs of the ER stress marker activating transcription factor 6 (ATF6; blue) and CC3 (red) colocalization with the neuronal marker NeuN (green) in the post‐mortem human OFC of individuals with AUD. White arrows indicate ATF6 + IR and CC3 + IR colocalization with NeuN. Scale bar = 20 μm

Preclinical studies report that NADPH oxidative stress is linked to ER stress and cell death.^17^ In the present study, we report AUD increases of NADPH oxidases, oxidative stress markers, ER stress‐associated molecules, and cell death caspases in the post‐mortem human OFC. To confirm reductions of neuron populations in the AUD OFC consistent with cell death, we assessed expression of the neuronal markers NeuN and MAP 2. Immunohistochemical assessment of the neuronal marker NeuN revealed a 24% (±4%) reduction in the AUD OFC, *t*(13.4) = 2.8, *p* = 0.014, Welch's *t* test (Figure 8A), relative to CONs. Expression of NeuN + IR neurons negatively correlates with expression of the executioner cell death caspase cleaved caspase 3 (*r* = −0.58, *p* = 0.008) and the ER stress‐associated apoptotic protease caspase 12 (*r* = −0.54, *p* = 0.015), consistent with neuronal degeneration in the AUD OFC (Figure 8B). Consistent with the NeuN + IR data, we report an AUD‐induced 26% (±7%) reduction of the neuronal marker MAP 2 in the AUD OFC, *t*(15) = 2.2, *p* = 0.043 (Figure 8C), relative to CONs. Expression of MAP 2 + IR neurons negatively correlates with cleaved caspase 3 (*r* = −0.58, *p* = 0.008) but not caspase 12 (*p* = 0.078; Figure 8D), further supporting neuronal cell death in the OFC of individuals with AUD. To investigate the role of NADPH oxidase (i.e., NOX2) and ER stress in AUD‐induced neuronal death in the OFC, triple immunofluorescence of either NOX2 or the ER stress proteins GRP78 and ATF6 in combination with cleaved caspase 3 and the neuronal marker NeuN was performed. Triple immunostaining revealed co‐expression of NOX2, GRP78, and ATF6 with cleaved caspase 3 and NeuN + IR neurons (Figure 9), consistent with NADPH oxidases and ER stress contributing to neuronal cell death. Further, expression of neuronal markers (i.e., NeuN and MAP 2) negatively correlates with expression of the majority of ER stress‐associated proteins (Figure 8E). Together, these data suggest that induction of NOX and ER stress markers contributes to chronic alcohol consumption‐induced neurodegeneration in human AUD brain.

## 4 DISCUSSION

**FIGURE 10:**
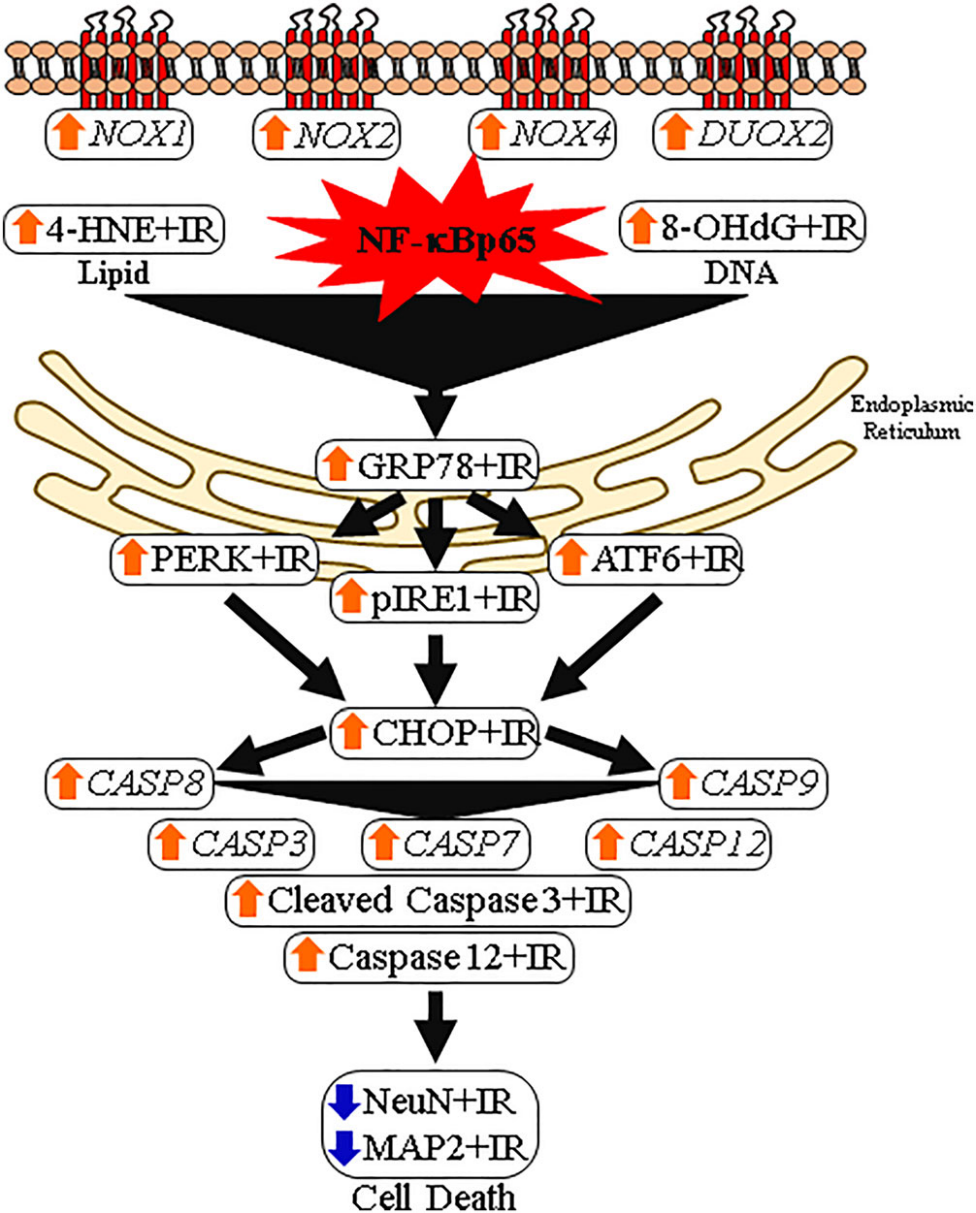
Induction of NADPH oxidative stress and endoplasmic reticulum (ER) stress contributes to neuronal cell death in alcohol use disorder (AUD). Simplified schematic of AUD induction of NADPH oxidative stress through activation of the proinflammatory nuclear transcription factor NF‐κB p65 leading to ER stress and cell death‐associated caspase cascades in the orbitofrontal cortex (OFC). Chronic heavy alcohol consumption causes activation of NF‐κB p65 in association with upregulation of NADPH oxidases (i.e., NOX1, NOX2, NOX4, and DUOX2) as well as the oxidative stress markers 4‐hydroxynonenal (4‐HNE), which identifies lipid peroxidation, and 8‐hydroxy‐2′‐deoxyguanosine (8‐OHdG), which identifies DNA oxidation. NADPH oxidases and oxidative stress markers contribute to ER stress^17^ through dissociation of glucose‐regulated protein 78 (GRP78) from ER resident transmembrane sensor proteins, including inositol requiring protein 1 (IRE1), protein kinase RNA‐like endoplasmic reticulum kinase (PERK), and activating transcription factor 6 (ATF6), which signal through the pro‐apoptotic transcription factor C/EBP homologous protein (CHOP).^27^ CHOP is a key cell death effector that promotes transcription of caspase genes^28^ including the ER stress‐associated apoptotic protease caspase 12^40^ and the executioner caspase cleaved caspase 3.^44^ These data implicate induction of oxidative stress and ER stress within the same cells as contributing to neuronal cell death in the post‐mortem human OFC of individuals with AUD.

To our knowledge, this is the first study to report upregulation of NAPDH oxidase and oxidative stress markers in association with activation of ER stress and caspase‐mediated cell death pathways in the post‐mortem human OFC of individuals with AUD. The RTPCR mRNA and IHC cellular data presented here complement and extend our prior AUD OFC studies describing upregulation of proinflammatory HMGB1‐TLR‐NFκB neuroimmune signalling cascades leading to neurodegeneration.^3^ Preclinical models link activation of proinflammatory nuclear transcription factor NF‐κB p65 (RELA) to generation of proinflammatory NADPH oxidases (i.e., NOX2) and reactive oxygen species.^1^, ^15^ Similar to our findings in the post‐mortem human OFC of individuals with AUD, induction of TLR4‐RELA in neurodegenerative disorders and other pathological conditions is associated with induction of oxidative stress, ER stress, and neuronal cell death.^41^, ^42^ In the present study, we report that AUD increased several members of the NADPH oxidase family (i.e., *NOX1*, *NOX2*, and *NOX4*) and the dual oxidase *DUOX2* as well as the oxidative stress lipid peroxidation marker 4‐HNE and the DNA oxidation marker 8‐OHdG. Expression of NADPH oxidases and oxidative stress markers (i.e., 4‐HNE and 8‐OHdG) positively correlate with levels of RELA, suggesting an association between activation of NF‐κB and generation of proinflammatory oxidases and ROS leading to oxidative stress in the AUD OFC. NADPH oxidase production of ROS has been implicated in induction of the UPR and ER stress,^17^ and we report an AUD‐induced increase in mRNA and protein expression of the master regulator protein GRP78/BiP, the ER stress‐associated transmembrane sensors ATF6, PERK, and pIRE1, and the pro‐apoptotic transcription factor CHOP in the post‐mortem OFC. Interestingly, NADPH oxidases and ROS are reported to promote ER stress activation of CHOP which results in cell death,^43^ consistent with our observations that NADPH oxidase/dual oxidases and oxidative stress markers (i.e., 4‐HNE and 8‐OHdG) positively correlate with ER stress‐associated markers. Further, we report co‐expression of NOX2 with ER stress markers, indicating activation of oxidative stress and ER stress within the same cells. CHOP is a key UPR pro‐apoptotic effector that promotes transcription of pro‐apoptotic genes^28^ and we report induction of cell death‐associated initiator and executioner caspase genes (*CASP3*, *CASP7*, *CASP8*, and *CASP9*), consistent with activation of protease cell death cascades in the post‐mortem OFC of individuals with AUD. Indeed, caspase 3 is an executioner caspase that drives the morphological and biochemical changes associated with apoptosis,^44^ and we report activated cleaved caspase 3 in the OFC positively correlates with ER stress‐associated proteins and co‐localizes with the NADPH oxidase NOX2, indicative of oxidative stress and ER stress contributing to cell death in AUD. Interestingly, we also report increased protein and mRNA expression of caspase 12, which is a unique endoplasmic reticulum pro‐apoptosis protease that participates in ER stress‐induced apoptosis.^45^ Expression of caspase 12 similarly positively correlates with expression of ER stress‐associated proteins and colocalizes with the NADPH oxidase NOX2. These data are consistent with concerted activation of oxidative stress and ER stress pathways within the same cell leading to cell death‐associated caspase cascades, which could contribute to the observed neuronal cell death in the OFC of individuals with AUD.^1^, ^3^, ^7^ Indeed, in a prior study, we reported increased colocalization of the neuronal marker NeuN with the cell death marker Fluoro‐Jade B in OFC tissue samples from the same individuals used in the present investigation indicative of increased neuronal cell death in the post‐mortem human OFC of individuals with AUD.^1^ Consistent with activation of this cell death cascade is our finding of reduced neuronal expression of NeuN and MAP 2 that negatively correlates with expression of cleaved caspase 3 and caspase 12 as well as expression of ER stress‐associated proteins. Taken together, these data suggest that AUD induces proinflammatory NADPH oxidase/oxidative stress and ER stress that could contribute to induction of pro‐apoptotic cell death cascades and neurodegeneration in the OFC (Figure 10).

Activation of proinflammatory neuroimmune signalling is a hallmark feature of AUD and neurodegenerative disorders.^2^, ^46^, ^47^ Our laboratory and others report upregulation of proinflammatory TLRs, the endogenous TLR agonist HMGB1, and downstream activation of the nuclear transcription factor RELA leading to cytokine and chemokine signalling in the post‐mortem OFC of individuals with AUD.^3^, ^7^, ^48^, ^49^, ^50^ In addition to driving proinflammatory cytokine and chemokine induction, activation and nuclear translocation of RELA induces transcription of NF‐κB subunits and proinflammatory genes, including NADPH oxidases and ROS.^15^, ^51^, ^52^ Preclinical models link ethanol‐ and proinflammatory TLR4‐induced activation of NF‐κB to generation of proinflammatory NADPH oxidases (i.e., NOX2) and ROS that is blocked in NOX2^−/−^ knockout mice as well as by treatment with the NOX inhibitor diphenyliodonium.^1^, ^16^ NADPH oxidase‐mediated oxidative stress is implicated in many neurological diseases,^53^, ^54^ but the role of AUD‐induced oxidative stress in the human central nervous system (CNS), with the exception of NOX2,^1^ is largely unknown. In the present study, we report induction of several members of the NADPH oxidase family (i.e., *NOX1*, *NOX2*, and *NOX4*), the dual oxidase *DUOX2*, and the oxidative lipid peroxidation marker 4‐HNE and the DNA oxidation marker 8‐OHdG in the AUD OFC. Expression of these oxidative stress markers positively correlates with RELA, consistent with an association between NF‐κB activation and oxidative stress. We previously reported AUD‐associated ROS generation, and NF‐κB and NOX2 expression in human OFC neurons as well as microglia and astrocytes,^1^, ^3^ consistent with NF‐κB activation contributing to NADPH oxidase generation of ROS across neurons and glia that culminates in neurodegeneration.^1^, ^7^

The RELA‐mediated induction of NADPH oxidases and subsequent generation of ROS could contribute to ER stress and the UPR.^17^ Chronic alcohol consumption and AUD‐induced activation of ER stress and the UPR may accelerate age‐related neurodegeneration observed in AD and PD. For example, we found increased expression of phosphorylated (activated) IRE1 in the AUD OFC, and IRE1 has been linked to AD‐associated pathogenesis.^55^ The ER is a dynamic cellular organelle responsible for the synthesis, folding, modification, and transport of proteins.^56^, ^57^ Ageing and pathological conditions increase protein‐folding demands and disrupt protein‐folding capacity culminating in an ER imbalance and accumulation of unfolded or misfolded proteins in the ER lumen.^58^ To re‐establish homeostasis and minimize cell damage, cells activate the unfolded protein response (UPR) whose function is to restore ER homeostasis^21^, ^22^ through dissociation of the master regulator protein GRP78/BiP^59^, ^60^ from ATF6, IRE1α, and PERK. These signalling sensors work to restore ER function through degradation of ER‐bound mRNAs, inhibition of protein translation, and formation of protein folding enzymes.^23^, ^24^ While transient ER stress can typically be resolved through enhancing the folding capacity of the ER,^25^ chronic long‐lasting ER stress that does not recover results in cellular dysfunction and cell death through activation of the C/EBP homologous protein CHOP.^26^ For instance, induction of the ER stress cascade in liver is initially neuroprotective, but prolonged or unresolved activation of ER stress signalling results in cell death.^30^ Our observations that AUD induces mRNA and protein expression of GRP78 and the ER stress‐associated transmembrane sensors ATF6, PERK, and pIRE1 as well as the pro‐apoptotic effector CHOP in the post‐mortem human OFC, suggests that AUD causes activation of the UPR. CHOP is one of the key UPR pro‐apoptotic effectors that promotes transcription of pro‐apoptotic genes^28^, ^61^, ^62^ including caspase 12, which is a unique endoplasmic reticulum apoptosis protease that participates in ER stress‐induced apoptosis.^45^ Our finding of induction of cell death‐associated caspase genes as well as cleaved caspase 3^7^ and caspase 12 in the AUD OFC supports ER stress‐associated cell death mechanisms as contributing to the neurodegeneration observed in AUD. Consistent with these data, we previously reported similar activation of caspase‐mediated cell death signalling pathways in the post‐mortem hippocampus of individuals with AUD.^63^ Activation of the CHOP pathway can also induce cell death through activation of Death Receptors 4/5 via TRAIL. We previously reported upregulation of TRAIL and Death Receptors 4/5 in the post‐mortem human OFC of individuals with AUD.^7^ These data suggest that AUD‐associated chronic alcohol consumption causes ER stress in the OFC, leading to activation of caspase‐associated cell death cascades contributing to neuronal cell death.

Oxidative stress, ER stress, and neurodegeneration are features of addiction and neurodegenerative disorders.^64^, ^65^, ^66^, ^67^, ^68^, ^69^ In AUD individuals, neuroimaging studies report diminished OFC volume and brain connectivity^11^ while post‐mortem histological studies report decreased OFC glial and neuronal densities that correlate with the duration of alcohol dependence in individuals with AUD.^9^ Consistent with these data is our observation of reductions in neuronal marker expression of NeuN and MAP 2, which negatively correlate with expression of the cell death‐associated proteases cleaved caspase 3 and caspase 12. While we find that AUD‐induced cell death in the post‐mortem human OFC primarily reflects an apoptotic cell death process,^7^, ^70^ there is also evidence of neurodegeneration as we previously reported NeuN colocalization with FluoroJade B.^1^ In preclinical studies, ethanol treatment similarly increases cortical neuronal cell death and caspase 3 activity in the mouse neocortex that is blocked by treatment with the NOX inhibitor diphenyliodonium.^1^ Similarly, NOX2^−/−^ deficiency in mice protects against TLR4 activation‐induced neuronal loss,^16^ implicating neuroimmune induction through TLRs in oxidative stress‐induced neurodegeneration. These findings are consistent with AUD progressively inducing persistent proinflammatory HMGB1‐TLR‐NFκB neuroimmune signalling that is accompanied by NADPH oxidase generation of ROS that results in ER stress, contributing to the modest and diffuse neurodegeneration associated with AUD that may sensitize the brain to age‐related neurodegeneration and disease.

In conclusion, we report induction of NADPH oxidases (i.e., *NOX1*, *NOX2*, *NOX4*), the dual oxidase *DUOX2*, and the oxidative stress markers 4‐HNE and 8‐OHdG in the post‐mortem human AUD OFC that positively correlate with upregulation of RELA, consistent with NF‐κB generation and induction of proinflammatory oxidases and ROS leading to oxidative stress. Induction of oxidative stress is accompanied by increased expression of the ER stress‐associated GRP78‐ATF6, pIRE1, and PERK‐CHOP signalling cascades that positively correlates with induced oxidases and oxidative stress markers and colocalizes with the NADPH oxidase NOX2, suggesting that AUD‐induced oxidative stress contributes to induction of ER stress in the human OFC. The observed activation of oxidative stress and ER stress signalling in the AUD OFC is accompanied by induction of cell death‐associated caspases and loss of neuronal markers (i.e., NeuN and MAP 2), consistent with activation of cell death cascades in the post‐mortem OFC of individuals with AUD. Expression of the executioner protease cleaved caspase 3 and the ER pro‐apoptotic protease caspase 12 positively correlate with ER stress‐associated proteins and colocalize with NOX2, consistent with coordinated activation of oxidative‐ER stress within the same cell leading to induction of cell death‐associated caspase cascades that could contribute to the observed AUD‐induced neuronal cell death in the OFC. These data implicate HMGB1‐TLR‐NFκB neuroimmune signalling and NADPH oxidase generation of ROS in induction of ER stress as contributing to neuronal cell death in the post‐mortem OFC of individuals with AUD.

## CONFLICT OF INTEREST

The authors report no conflict of interest.

## AUTHOR CONTRIBUTIONS

LQ, RPV, and FTC were responsible for the study concept and design, data analysis and interpretation of findings, and drafting of the manuscript. All authors critically reviewed content and approved the final version for publication.

## Data Availability

The data that support the findings of this study are available from the corresponding author upon reasonable request.
